# Undilatable Stent Neoatherosclerosis Treated with Ad Hoc Rotational Atherectomy

**DOI:** 10.1155/2017/3168067

**Published:** 2017-01-10

**Authors:** Michael Koutouzis, Maria Agelaki, Christos Maniotis, Ioannis Tsiafoutis, Vasileios Patris, Mihalis Argyriou

**Affiliations:** ^1^Cardiology Department, Hellenic Red Cross Hospital of Athens, Athens, Greece; ^2^Cardiac Surgery Department, Evaggelismos General Hospital, Athens, Greece

## Abstract

A middle age woman with known ischemic heart disease and old stents in proximal left anterior descending coronary artery (LAD) was admitted to Coronary Care Unit with acute coronary syndrome. The coronary angiography showed one vessel disease with significant restenosis within the previously implanted stents. The lesion was tough and remained undilatable despite high pressure balloon inflation. Eventually, the balloon ruptured creating a massive dissection of the LAD beginning immediately after the distal part of the undilatable lesion. We proceeded with a challenging ad hoc rotational atherectomy of the lesion and finally stenting of the lesion. In-stent restenosis many years after stent implantation is considered to be mainly due to neoatheromatosis compared to intimal hyperplasia, making lesion treatment more difficult and unpredictable.

## 1. Introduction

In-stent stenosis long time after initial stent implantation is not an uncommon finding in our days, but treatment of this type of lesion, especially under unstable conditions, remains a challenge. Our recent knowledge about the histology of these lesions, being comprised mainly of neoatheroma and not neointima hyperplasia, explains the need for different management approach and the high rate of serious complications, like dissection.

## 2. Case

A 55-year-old female with a known history of ischemic heart disease, hypertension, dyslipidemia, and insulin dependent diabetes mellitus presented to the Accident and Emergency Department of our hospital with new onset chest pain. The patient had a successful percutaneous coronary intervention in the proximal part of LAD for stable angina 9 years ago and two drug eluting stents (DES) (Taxus Liberte, Boston Scientific) were uneventfully implanted then. On admission, the electrocardiogram showed no significant changes and the cardiac troponin was slightly elevated. The patient was admitted to the Cardiac Intensive Care unit with the diagnosis of non-ST elevation myocardial infarction. The initial cardiac ultrasound showed normal ejection fraction with no regional wall motion abnormalities and no valvular abnormalities. The patient remained symptomatic with chest pain recurrences after maximum antianginal treatment with intravenous nitroglycerin and b-blockers, so we decided to proceed to emergency coronary angiography.

**Figure 1:**
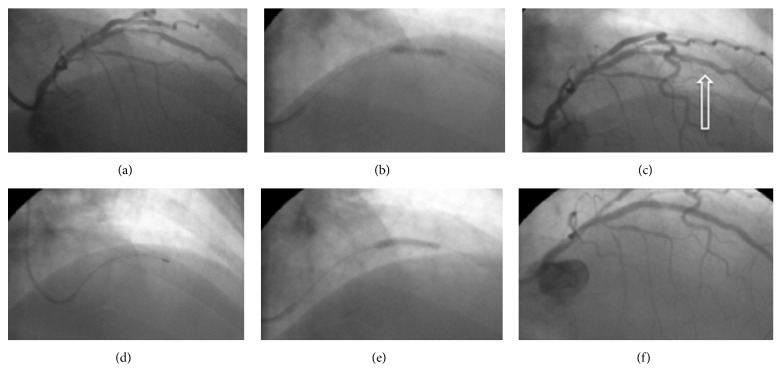
The angiographic presentation of the course of the events. Images are captured at the right anterior oblique view. (a) The initial cardiac catheterization showing severe in-stent stenosis, quite tight at the proximal part of the stent involving the ostium of the diagonal branch. (b) Balloon expansion at high pressure failed to expand the lesion. (c) After balloon rupture, there was a massive type B coronary artery dissection starting at the distal part of balloon expansion (the radiolucent lines are highlighted with the arrow). (d) Rotational atherectomy was successfully performed with a 1.25 mm burr passing through the critical stenosis. (e) Successful balloon expansion after rotablation. (f) Excellent final result with TIMI III flow after stent deployment.

The coronary angiography was performed through the right radial approach after placement of a 6 Fr radial sheath (KDL, China) and it showed one vessel disease with significant restenosis within the previously implanted stents (Figure 1(a)). We decided to proceed to ad hoc percutaneous coronary intervention within the restenotic proximal LAD lesion. The patient was loaded with 60 mg prasugrel in addition to the already taken aspirin and intravenous bivalirudin was administered for anticoagulation at 0.75 mg/kg bolus followed by an infusion of 1.75 mg/kg/hr. A JL 3.5 6 F guide catheter was exchanged over a 0.035 guidewire and it was placed in the ostium of the left main. We crossed the lesion using a workhorse guidewire (Luge, Boston Scientific) and dilatation was performed with a 2.0 mm semicompliant balloon (Sprinter Legend RX, Medtronic), but the lesion remained undilatable (Figure 1(b)). At the third attempt to expand the lesion using high pressure (at 22 Atm), the balloon ruptured producing a massive dissection of the LAD beginning immediately after the distal part of the undilatable lesion (Figure 1(c)). The presence of radiolucent area during contrast injection suggesting the creation of double lumen without persistence after dye clearance classified the dissection as type B according to the NHLBI classification [1]. The patient started complaining of chest pain and the ECG monitor showed ST segment elevation in lead V1.

The placement of a stent distal to the undilatable lesion was attempted unsuccessfully, due to inability to pass the lesion. We did not insist further on in-stent placement with other options, like guide extension catheters, because even if we succeeded the patient would still be in need of coronary artery bypass grafting (CABG) operation, due to the undilatable proximal stenosis. The need for satisfactory lesion dilation was a prerequisite before any other action. Expansion of the stenosis with a cutting balloon failed completely as it did not even manage to go through the critical part of the lesion. In the meantime, the patient was still in pain and she became haemodynamically unstable making inotropic support necessary. Three options were available at this time point:

- Proceeding with an emergency CABG while on full antiplatelet treatment with prasugrel
- Treating the dissection with prolonged balloon inflation
- Proceeding to rotational atherectomy and stenting

The first option was rejected initially due to the very high bleeding risk of the patient and it was reserved as a last resort. Balloon inflation at the site of dissection after so many attempts for dilation was considered very venturous as it could easily augment the dissection even further, making surgical solution inevitable. Moreover, complete vessel occlusion could potentially deteriorate left ventricular function even further and induce a haemodynamic collapse. Taking into account the above-mentioned parameters, we decided to proceed to ad hoc rotational atherectomy, even though coronary dissection is considered as a contraindication for this technique [2]. A 1.25 × 15 mm over-the-wire balloon (Sprinter OTW, Medtronic) was placed at the distal part of the LAD, the Luge guidewire was removed, and a Floppy Rotawire was placed at the distal part of LAD. Rotational atherectomy was successfully performed with a 1.25 mm burr (Figure 1(d)), but the lesion was still undilatable with a 2 mm semicompliant balloon. We upgraded the burr to a 1.5 mm and successfully rotablated the lesion once more. Dilation was performed using 2.0 mm and 2.5 mm semicompliant balloons (Figure 1(e)) (Sprinter Legend, Medtronic) and the dissected and the previously undilated segment were stented. Three DES were placed in a row starting distally and moving proximally (Resolute Integrity, Medtronic) with a favorable angiographic result (Figure 1(f)). The patient was pain-free at the end of the procedure and a modest troponin I elevation was observed the next day. There was no evidence of left ventricular dysfunction at the echocardiogram performed the next day. The patient was discharged after three days with dual antiplatelet treatment (aspirin 100 mg daily and prasugrel 10 mg daily) and she remains asymptomatic three months after the procedure.

## 3. Discussion

This is an interesting case report that highlights the challenges one can confront when treating old in-stent lesions. In-stent restenosis (ISR) through the neointimal formation was thought so far to be a major reason for coronary lumen narrowing after stent deployment. Both histology and physical history of ISR differ among bare metal stents (BMS) and drug eluting stents (DES). In DES, the neointima consists mainly of proteoglycan matrix and to a lower extent, of vascular smooth muscle cells, showing a late catch-up at almost 2 years from implantation [3]. On top of that, new atherosclerotic changes happen due to the chronic inflammation and the incomplete endothelialization of the stent struts. The drug eluting stents are more prone to neoatheroma formation, as this process appears to be more frequent and it takes place earlier compared to BMS. These lesions follow the well-studied course of plaque rupture when they become unstable leading to late or very late stent thrombosis [4]. The effect of in-stent neoatherosclerosis on prognosis is significant, as there is an association with increased major adverse cardiac events. The presence of high levels of low density lipoprotein and C-reactive protein are the major predictors of neoatheroma formation. Time from stent implantation is another crucial and easily identifiable parameter [5]. Despite the different time course of neoatherosclerosis in BMS and DES, when time from implantation exceeds 5 years, then the lesions found are mainly of neoatheroma rich in cholesterol, necrotizing foam cell, and inflammation [6].

Nevertheless, the lesion seen during angiography is difficult to distinguish between the usually dilatable in-stent restenosis and the tough stent neoatherosclerosis. The time of intervention after the initial procedure raises the suspicion for the presence of neoatheroma, but angiography has limitations in lesion characterization and it is of a little help. Advanced intracoronary imaging with virtual histology intravascular ultrasonography (IVUS) and optical coherence tomography (OCT) provide high resolution images and they are useful tools for the diagnosis and the treatment especially in complicated cases as in ours [4]. Signal interference from the metal struts of the old stent makes IVUS less efficient in neointima tissue characterization compared to the ultrahigh resolution OCT. Unfortunately, either modality was not an option in our case as the patient became symptomatic and haemodynamically unstable just after the dissection. Time was crucial and we decided to continue treatment based on angiographic criteria only.

The presence of neoatherosclerosis makes the lesion tough, difficult to dilate, and more prone to complications like dissection. When the dissection ensues, the risk of complete occlusion of the vessel is high and sealing of the dissection with stents becomes mandatory. When dissection happens and still the stenosis remains critical, then the treatment options are limited. Options other than rotablation, like the cutting wire technique or the high pressure dilation, are usually ineffective and they have an inherent risk of dissection expansion. Even though conservative management of the dissection for 4 weeks is proposed before applying rotablation, in our case the patient was symptomatic with ongoing ACS and immediate action was necessary. Rotational atherectomy with small burr size with minimum advancement and high speed seems to be safe and efficient for the treatment of undilated calcified lesions complicated by dissection [2]. In our case, two sessions of rotablation with increasing burr size was enough to open up the lesion and allow full expansion subsequently with a balloon. The procedure was completed uneventfully, leading to symptoms quiescence and only minimum troponin elevation.

## 4. Conclusion

In-stent restenosis long time after primary intervention, usually more than five years, should raise suspicion for the presence of neoatherosclerosis. Such a knowledge is crucial in planning management of tight chronic lesions in order to avoid complications like dissection.
